# Supplementary material for: Differential contributions of left-hemispheric language regions to basic semantic composition
Source: Brain Struct Funct. 2021 Jan 30;226(2):501–18. doi: 10.1007/s00429-020-02196-2 (PMC7910266; doi:10.1007/s00429-020-02196-2)
Supplement: Supplementary file 1 — Electronic supplementary material 1 (DOCX 130 kb) [file 429_2020_2196_MOESM1_ESM.docx]

**Supplementary Material**

**Supplementary Materials and Methods**

**Supplementary Table 1.** Psycholinguistic variables for the two real-word conditions.

|  | Anomalous | Meaningful | *p* |
| --- | --- | --- | --- |
| **Adjectives** | | | |
| Frequency | 2.13 (0.96) | 2.11 (0.95) | 0.900 |
| OLD-20 | 1.98 (0.45) | 1.91 (0.43) | 0.400 |
| **Nouns** | | | |
| Frequency | 2.57 (0.59) | 2.57 (0.55) | 0.960 |
| OLD-20 | 1.66 (0.35) | 1.67 (0.31) | 0.830 |
| Concreteness | 4.86 (0.13) | 4.85 (0.15) | 0.720 |
| **Pairs** |  |  |  |
| Meaningfulness rating | 1.47 (0.31) | 5.49 (0.36) | **< 0.0001** |
|  | *1.34 (0.24)* | *5.69 (0.27)* |  |

Frequency and OLD-20 (orthographic neighborhood) measures were taken from the SUBTLEX-DE database and frequency is given as log-transformed per 1 million words. Concreteness was determined using concreteness ratings for 40.000 English words. Ratings were obtained from 20 participants who did not take part in the fMRI experiment. Ratings in italics are averaged ratings of the fMRI participants in a post-hoc questionnaire. They indicate great overlap with our predefined conditions. Numbers in brackets represent standard deviation.

**Supplementary Table 2.** Stimulus list for meaningful and anomalous phrases

| **Condition** | **Stimulus (english translation)** | **Mean Plausibility Rating** |
| --- | --- | --- |
| **A** | nervoeser Salat (anxious salad) | 1.05 |
| **A** | aengstliches Holz (anxious wood) | 1.1 |
| **A** | braver Lappen (obedient cloth) | 1.1 |
| **A** | felsiges Schaf (rocky sheep) | 1.1 |
| **A** | fluessiges Auto (liquid car) | 1.1 |
| **A** | gestimmtes Schwein (tuned pig) | 1.1 |
| **A** | ruhiger Krug (quiet jug) | 1.1 |
| **A** | stumpfer Fisch (blunt fish) | 1.1 |
| **A** | zahmes Brett (tame board) | 1.1 |
| **A** | koestliches Schiff (delicious ship) | 1.2 |
| **A** | morsches Kamel (rotten camel) | 1.2 |
| **A** | toter Korb (dead basket) | 1.2 |
| **A** | blinder Finger (blind finger) | 1.25 |
| **A** | frisches Dreieck (fresh triangle) | 1.25 |
| **A** | hoelzerner Schaum (wooden foam) | 1.25 |
| **A** | lautes Auge (loud eye) | 1.25 |
| **A** | modischer Spinat (fashinable spinach) | 1.25 |
| **A** | mutiger Knochen (brave bone) | 1.25 |
| **A** | poroeser Schwan (porose swan) | 1.25 |
| **A** | sportliches Glas (sporty glass) | 1.25 |
| **A** | stolzes Kabel (proud wire) | 1.25 |
| **A** | pikanter Teppich (spicy carpet) | 1.3 |
| **A** | behaarter Ofen (hairy oven) | 1.35 |
| **A** | defekter Loewe (defective lion) | 1.35 |
| **A** | hungriges Klavier (hungry piano) | 1.35 |
| **A** | luftiger Teller (airy plate) | 1.35 |
| **A** | ovales Pferd (oval horse) | 1.35 |
| **A** | eckiges Pferd (rectangular horse) | 1.4 |
| **A** | lockerer Pfau (loose peacock) | 1.45 |
| **A** | niedriger Pinsel (low brush) | 1.45 |
| **A** | defekter Fuchs (defective fox) | 1.5 |
| **A** | einsamer Motor (lonely engine) | 1.5 |
| **A** | leerer Guertel (empty belt) | 1.5 |
| **A** | senkrechter Helm (vertical helmet) | 1.5 |
| **A** | wilder Koffer (wild suitcase) | 1.5 |
| **A** | zackiger Schlauch (pointed tube) | 1.5 |
| **A** | biegsamer Mond (flexible moon) | 1.55 |
| **A** | faules Kleid (lazy dress) | 1.55 |
| **A** | junger Kittel (young smock) | 1.55 |
| **A** | bequemer Zahn (comfortable tooth) | 1.6 |
| **A** | gesunder Spiegel (healthy mirror) | 1.6 |
| **A** | wilder Stuhl (wild chair) | 1.6 |
| **A** | dummer Schal (stupid scarf) | 1.65 |
| **A** | stabiler Wolf (stable wolf) | 1.65 |
| **A** | weiblicher Tisch (female table) | 1.7 |
| **A** | freches Paket (naughty package) | 1.8 |
| **A** | kaputter Schwan (broken swan) | 1.85 |
| **A** | wachsames Plakat (vigilant poster) | 1.85 |
| **A** | sauberer Mond (clean moon) | 1.9 |
| **A** | unscharfes Hemd (blurry shirt) | 1.9 |
| **A** | offenes Fahrrad (open bicycle) | 1.95 |
| **A** | steinerner Brief (stone letter) | 1.95 |
| **A** | stoerrischer Berg (stubborn mountain) | 2.05 |
| **A** | schmales Herz (narrow heart) | 2.1 |
| **A** | eiserner Pilz (iron mushroom) | 2.2 |
| **A** | rostiger Vogel (rusty bird) | 2.25 |
| **M** | niedriger Spiegel low mirrow) | 4.55 |
| **M** | ruhiger Motor (quiet engine) | 4.65 |
| **M** | einsamer Vogel (lonely bird) | 4.75 |
| **M** | felsiger Berg (rocky mountain) | 4.85 |
| **M** | ovaler Teller (oval plate) | 4.85 |
| **M** | spitzes Dreieck (pointy triangle) | 4.95 |
| **M** | braves Schaf (obedient sheep) | 5.1 |
| **M** | senkrechter Pfosten (vertical pole) | 5.1 |
| **M** | gesundes Holz (healthy wood) | 5.15 |
| **M** | pikanter Fisch (spicy fish) | 5.2 |
| **M** | stolzer Pfau (proud peacock) | 5.2 |
| **M** | zackiger Stern (ponted star) | 5.2 |
| **M** | poroeser Knochen (porose bone) | 5.25 |
| **M** | steinernes Kreuz (stone cross) | 5.25 |
| **M** | toter Wolf (dead wolf) | 5.25 |
| **M** | defekter Schlauch (defective tube) | 5.3 |
| **M** | hohler Zahn (hollow tooth) | 5.3 |
| **M** | hoelzernes Schiff (wooden ship) | 5.35 |
| **M** | zahmer Loewe (tame lion) | 5.35 |
| **M** | aengstliches Pferd (anxious horse) | 5.4 |
| **M** | frischer Lappen (clean cloth) | 5.4 |
| **M** | eiserner Ofen (iron oven) | 5.45 |
| **M** | frecher Affe (naughty monkey) | 5.5 |
| **M** | morscher Baum (rotten tree) | 5.5 |
| **M** | sturer Esel (stubborn donkey) | 5.5 |
| **M** | handliches Paket (compact package) | 5.55 |
| **M** | hungriger Fuchs (hungry fox) | 5.55 |
| **M** | koestlicher Salat (delicious salad) | 5.55 |
| **M** | leeres Plakat (blank poster) | 5.55 |
| **M** | luftiges Kleid (airy dress) | 5.55 |
| **M** | weiblicher Schwan (female swan) | 5.55 |
| **M** | biegsames Kabel (flexible wire) | 5.6 |
| **M** | dummes Schaf (stupid sheep) | 5.6 |
| **M** | lautes Auto (loud car) | 5.6 |
| **M** | offener Beutel (open bag) | 5.6 |
| **M** | duenner Pinsel (thin brush) | 5.65 |
| **M** | krummer Finger (crooked finger) | 5.65 |
| **M** | blindes Auge (blind eye) | 5.7 |
| **M** | giftiger Pilz (poiseneous mushroom) | 5.7 |
| **M** | schmaler Guertel (narrow belt) | 5.7 |
| **M** | rostiges Schwert (rusty sword) | 5.8 |
| **M** | sauberes Glas (clean glass) | 5.8 |
| **M** | bequemes Hemd (comfortable shirt) | 5.85 |
| **M** | junger Hirsch (young deer) | 5.85 |
| **M** | leerer Koffer (empty suitcase) | 5.85 |
| **M** | roher Spinat (raw spinach) | 5.85 |
| **M** | runder Mond (round moon) | 5.85 |
| **M** | stabiles Regal (stable shelf) | 5.85 |
| **M** | blutiges Knie (bloody knee) | 5.9 |
| **M** | eckiger Tisch (rectangular table) | 5.9 |
| **M** | gestimmtes Klavier (tuned piano) | 5.9 |
| **M** | kaputtes Fahrrad (broken bicycle) | 5.9 |
| **M** | reifer Kaese (mature cheese) | 5.9 |
| **M** | modischer Schal (fashionable scarf) | 5.95 |
| **M** | bequemer Stuhl (comfortable chair) | 6 |
| **M** | unscharfes Foto (blurry photo) | 6 |

A = Anomalous, M = Meaningful. Ratings were obtained from 20 participants who did not take part in the fMRI experiment.

**Supplementary Results**


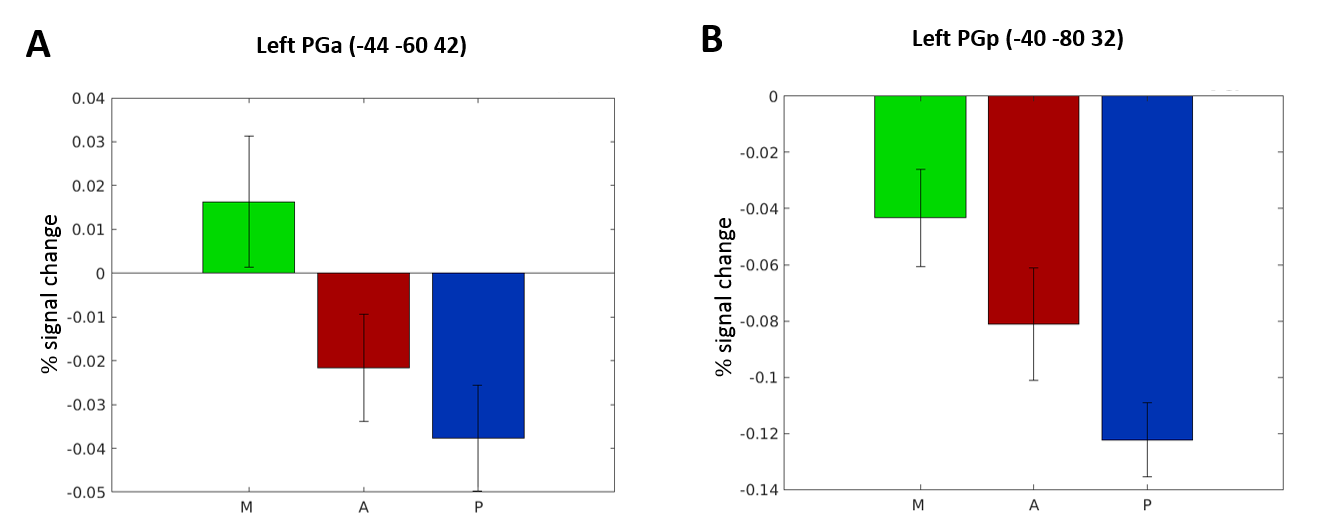


**Supplementary Figure 1**. Percent signal change for PGa (A) and PGp (B) for each condition compared to the implicit rest baseline. 6-mm spheres centered on the peak voxel from the contrast meaningful > anomalous for PGa and from meaningful > pseudowords for PGp were created using the MarsBaR toolbox (version 0.44; <http://marsbar.sourceforge.net/>).

**Supplementary Table 3.** Behavioral results.

|  | | **IMPLICIT** | | | | | **EXPLICIT** | | | |
| --- | --- | --- | --- | --- | --- | --- | --- | --- | --- | --- |
| **Predictor** | *Coef. ß* | | *SE(ß)* | *T* | *p* | *Coef. ß* | | *SE(ß)* | *T* | *p* |
| **Accuracy** |  | |  |  |  |  | |  |  |  |
| m > a | 1.02 | | 0.23 | 4.42 | **< 0.001** | -0.09 | | 0.18 | -0.49 | 0.62 |
| m > p | 1.14 | | 0.23 | 4.97 | **< 0.001** | -0.59 | | 0.19 | -3.08 | **< 0.01** |
| a > p | 0.12 | | 0.2 | 0.59 | 0.556 | -0.51 | | 0.19 | -2.6 | **< 0.01** |
| **Reaction Time** | | | | | | | | | | |
| m > a | 76.32 | | 4.28 | 17.84 | **< 0.001** | 67.99 | | 3.82 | 17.8 | **< 0.001** |
| m > p | 204.44 | | 5.14 | 39.76 | **< 0.001** | -8.59 | | 3.6 | -2.39 | **< 0.05** |
| a > p | 128.12 | | 6.53 | 19.61 | **< 0.001** | -76.58 | | 4.23 | -18.1 | **< 0.001** |

Parameter Estimates for the Fixed Effects with meaningful and anomalous as the reference contrasts for the pairwise comparisons in the Linear Mixed-Effects Models. m = meaningful, a = anomalous, p = pseudowords.

**Activation Tables**

Activation maps were thresholded at p < .05 FDR-corrected. Up to 5 peaks per cluster that are more than 8 mm apart are reported.

For the psychophysiological interaction, Contrast images were thresholded at p < 0.05, cluster-level family wise error (FWE) corrected, with a voxel-wise threshold of p < 0.001. L = left, R = right, ACC = anterior cingulate cortex, AG = angular gyrus, ATL = anterior temporal lobe, IFG = inferior frontal gyrus, ITG = inferior temporal gyrus, IPS = intraparietal sulcus, SMA = supplementary motor area, SMG = supramarginal gyrus, M1 = primary motor cortex, MTG = middle temporal gyrus, PFC = prefrontal cortex, MCC = middle cingulate cortex, v = ventral, d = dorsal, m = medial, p = posterior, a = anterior.

**Supplementary Table 4**. Activation peaks for meaningful > anomalous phrases in the explicit task

| **Region** | **Cluster size (voxels/mm³)** | **x** | **y** | **z** | **T** |
| --- | --- | --- | --- | --- | --- |
| **L and R ACC, DMPFC** | 328/ 5125 |  |  |  |  |
| L ACC |  | -2 | 48 | 5 | 5.91 |
| L superior medial gyrus |  | 0 | 58 | 10 | 5.42 |
| L ACC |  | -2 | 36 | 8 | 4.9 |
| L superior medial gyrus |  | 0 | 46 | 20 | 4.84 |
| R ACC |  | 3 | 33 | 15 | 4.5 |
| **L AG, IPS, SMG (PF/PFm)** | 286/4468 |  |  |  |  |
| L AG (PGa/PFm) |  | -44 | -60 | 42 | 5.59 |
| L SMG (PF) |  | -57 | -42 | 48 | 5.36 |
| L AG (PGa) |  | -44 | -54 | 55 | 5.26 |
| L IPS (hIP3) |  | -34 | -50 | 50 | 4.88 |
| L AG (PGa) |  | -42 | -70 | 42 | 4.72 |
| **L pMTG** | 90/1406 |  |  |  |  |
| L pMTG |  | -60 | -32 | -15 | 5.11 |
| L pMTG |  | -60 | -52 | -8 | 4.45 |
| L pMTG |  | -57 | -44 | -10 | 4.17 |
| **L vmPFC** | 82/1281 |  |  |  |  |
| L superior orbital gyrus |  | -12 | 18 | -20 | 6.25 |
| L rectal gyrus (s32) |  | -7 | 28 | -18 | 5 |
| L rectal gyrus |  | -14 | 30 | -15 | 4.53 |
| **R AG** | 42/656 |  |  |  |  |
| R AG (PGp) |  | 40 | -70 | 38 | 5.19 |
| R middle occipital gyrus |  | 43 | -77 | 32 | 4.68 |
| **R caudate nucleus** | 24/375 |  |  |  |  |
| R caudate nucleus |  | 8 | 13 | 8 | 6.14 |
| **L MCC** | 20/312 |  |  |  |  |
| L MCC |  | -4 | -22 | 40 | 4.36 |

**Supplementary Table 5.** Activation peaks for meaningful > pseudoword phrases in the explicit task

| \| **Region** \| **Cluster size (voxels/mm ³)** \| **x** \| **y** \| **z** \| **T** \| \| --- \| --- \| --- \| --- \| --- \| --- \| \| **L aIFG, DMPFC, vmPFC** \| 6400/100000 \|  \|  \|  \|  \| \| L middle orbital gyrus \|  \| -47 \| 48 \| -8 \| 7.65 \| \| L aIFG (pars orbitalis) \|  \| -47 \| 40 \| -12 \| 7.05 \| \| L aIFG (pars orbitalis) \|  \| -34 \| 36 \| -12 \| 6.88 \| \| L aIFG (pars orbitalis) \|  \| -32 \| 20 \| -18 \| 6.79 \| \| L superior medial gyrus \|  \| -2 \| 46 \| 42 \| 6.61 \| \| **L AG, pSMG, IPS** \| 1879/29359 \|  \|  \|  \|  \| \| L AG (PGp) \|  \| -40 \| -80 \| 32 \| 6.59 \| \| L AG (PGp) \|  \| -47 \| -70 \| 28 \| 6.22 \| \| L AG (PGa) \|  \| -44 \| -60 \| 42 \| 5.78 \| \| L AG \|  \| -40 \| -57 \| 25 \| 5.58 \| \| L AG (PGp) \|  \| -52 \| -64 \| 20 \| 5.23 \| \| **L pMTG/ITG/ATL** \| 1186/18531 \|  \|  \|  \|  \| \| L pMTG \|  \| -57 \| -50 \| -8 \| 7.35 \| \| L pITG \|  \| -60 \| -42 \| -12 \| 7.27 \| \| L pITG \|  \| -54 \| -57 \| -12 \| 7.01 \| \| L pMTG \|  \| -64 \| -32 \| -15 \| 5.43 \| \| L ATL \|  \| -52 \| -2 \| -38 \| 5.12 \| \| **R cerebellum** \| 887/13859 \|  \|  \|  \|  \| \| R cerebellum (crus I) \|  \| 40 \| -74 \| -42 \| 6.72 \| \| R cerebellum (crus II) \|  \| 18 \| -84 \| -38 \| 6.02 \| \| R cerebellum (crus I) \|  \| 13 \| -80 \| -25 \| 5.84 \| \| R cerebellum (crus I) \|  \| 36 \| -77 \| -35 \| 5.5 \| \| R cerebellum (crus I) \|  \| 43 \| -72 \| -35 \| 5.43 \| \| **R M1, premotor, somatosensory cortex** \| 274/4812 \|  \|  \|  \|  \| \| R primary somatosensory cortex \|  \| 36 \| -27 \| 48 \| 4.28 \| \| R primary motor cortex \|  \| 38 \| -20 \| 50 \| 4.17 \| \| R primary motor cortex \|  \| 33 \| -12 \| 52 \| 3.88 \| \| R primary somatosensory cortex \|  \| 48 \| -22 \| 60 \| 3.59 \| \| R primary somatosensory cortex \|  \| 50 \| -17 \| 48 \| 3.54 \| \| **R insula, temporal pole** \| 219/3421 \|  \|  \|  \|  \| \| R insula \|  \| 38 \| 23 \| -8 \| 6.4 \| \| R temporal pole \|  \| 40 \| 10 \| -20 \| 4.33 \| \| R temporal pole \|  \| 28 \| 8 \| -20 \| 3.87 \| \| R temporal pole \|  \| 30 \| 3 \| -12 \| 3.26 \| \| **L/R ACC** \| 128/2000 \|  \|  \|  \|  \| \| L ACC \|  \| -4 \| 6 \| 28 \| 4.54 \| \| R ACC \|  \| 8 \| 8 \| 28 \| 4.3 \| \| **R cerebellum** \| 109/1703 \|  \|  \|  \|  \| \| R cerebellum (lobule IX) \|  \| 3 \| -57 \| -48 \| 5.44 \| \| R cerebellum (lobule IX) \|  \| 10 \| -50 \| -40 \| 4.98 \| \| R cerebellum (lobule IX) \|  \| 20 \| -47 \| -40 \| 3.68 \| \| **L PCC/MCC** \| 87/1359 \|  \|  \|  \|  \| \| L PCC \|  \| -2 \| -42 \| 32 \| 3.64 \| \| L MCC \|  \| -7 \| -27 \| 38 \| 3.5 \| \| L MCC \|  \| 0 \| -34 \| 35 \| 3.38 \| \| **brain stem/thalamus** \| 77/1203 \|  \|  \|  \|  \| \| brain stem \|  \| -2 \| -34 \| -35 \| 4.63 \| \| brain stem \|  \| 0 \| -24 \| -35 \| 3.36 \| \| brain stem \|  \| -10 \| -40 \| -40 \| 3.3 \| \| brain stem \|  \| 13 \| -30 \| -35 \| 3.26 \| \| brain stem \|  \| 6 \| -27 \| -42 \| 2.93 \| \| **L cerebellum** \| 57/890 \|  \|  \|  \|  \| \| L cerebellum (crus II) \|  \| -34 \| -74 \| -40 \| 4.16 \| \| L cerebellum (crus I) \|  \| -27 \| -70 \| -32 \| 3.39 \| \| **R cerebellum** \| 55/859 \| 3 \| -57 \| -28 \| 4.43 \| \| **L precuneus** \| 52/812 \| -2 \| -54 \| 12 \| 4.61 \| \| **L insula** \| 46/718 \|  \|  \|  \|  \| \| L insula \|  \| -42 \| -2 \| 8 \| 4.27 \| \| L insula \|  \| -40 \| -4 \| 0 \| 3.39 \| \| **R fusiform, cerebellum** \| 45/703 \|  \|  \|  \|  \| \| R cerebellum (lobule IV-V) \|  \| 23 \| -32 \| -22 \| 3.62 \| \| R fusiform gyrus \|  \| 28 \| -42 \| -18 \| 3.6 \| \| R parahippocampal gyrus \|  \| 36 \| -37 \| -12 \| 3.19 \| \| **R posterior-medial frontal gyrus** \| 43/671 \| 10 \| -7 \| 55 \| 4.55 \| \| **L hippocampus** \| 42/656 \|  \|  \|  \|  \| \| L hippocampus \|  \| -30 \| -12 \| -12 \| 4.29 \| \| **R fusiform gyrus** \| 41/640 \|  \|  \|  \|  \| \| R fusiform gyrus \|  \| 38 \| -17 \| -25 \| 4.36 \| \| **R pITG** \| 29/453 \|  \|  \|  \|  \| \| R pITG \|  \| 56 \| -47 \| -12 \| 3.44 \| \| **R insula** \| 26/406 \|  \|  \|  \|  \| \| R insula \|  \| 40 \| -7 \| 0 \| 3.52 \| \| R insula \|  \| 40 \| 6 \| 0 \| 3.22 \| \| **R middle orbital gyrus** \| 23/359 \| 33 \| 40 \| -10 \| 4 \| \| **R AG (PGp)** \| 23/359 \| 53 \| -70 \| 25 \| 3.62 \| |
| --- | --- | --- | --- | --- | --- | --- | --- | --- | --- | --- | --- | --- | --- | --- | --- | --- | --- | --- | --- | --- | --- | --- | --- | --- | --- | --- | --- | --- | --- | --- | --- | --- | --- | --- | --- | --- | --- | --- | --- | --- | --- | --- | --- | --- | --- | --- | --- | --- | --- | --- | --- | --- | --- | --- | --- | --- | --- | --- | --- | --- | --- | --- | --- | --- | --- | --- | --- | --- | --- | --- | --- | --- | --- | --- | --- | --- | --- | --- | --- | --- | --- | --- | --- | --- | --- | --- | --- | --- | --- | --- | --- | --- | --- | --- | --- | --- | --- | --- | --- | --- | --- | --- | --- | --- | --- | --- | --- | --- | --- | --- | --- | --- | --- | --- | --- | --- | --- | --- | --- | --- | --- | --- | --- | --- | --- | --- | --- | --- | --- | --- | --- | --- | --- | --- | --- | --- | --- | --- | --- | --- | --- | --- | --- | --- | --- | --- | --- | --- | --- | --- | --- | --- | --- | --- | --- | --- | --- | --- | --- | --- | --- | --- | --- | --- | --- | --- | --- | --- | --- | --- | --- | --- | --- | --- | --- | --- | --- | --- | --- | --- | --- | --- | --- | --- | --- | --- | --- | --- | --- | --- | --- | --- | --- | --- | --- | --- | --- | --- | --- | --- | --- | --- | --- | --- | --- | --- | --- | --- | --- | --- | --- | --- | --- | --- | --- | --- | --- | --- | --- | --- | --- | --- | --- | --- | --- | --- | --- | --- | --- | --- | --- | --- | --- | --- | --- | --- | --- | --- | --- | --- | --- | --- | --- | --- | --- | --- | --- | --- | --- | --- | --- | --- | --- | --- | --- | --- | --- | --- | --- | --- | --- | --- | --- | --- | --- | --- | --- | --- | --- | --- | --- | --- | --- | --- | --- | --- | --- | --- | --- | --- | --- | --- | --- | --- | --- | --- | --- | --- | --- | --- | --- | --- | --- | --- | --- | --- | --- | --- | --- | --- | --- | --- | --- | --- | --- | --- | --- | --- | --- | --- | --- | --- | --- | --- | --- | --- | --- | --- | --- | --- | --- | --- | --- | --- | --- | --- | --- | --- | --- | --- | --- | --- | --- | --- | --- | --- | --- | --- | --- | --- | --- | --- | --- | --- | --- | --- | --- | --- | --- | --- | --- | --- | --- | --- | --- | --- | --- | --- | --- | --- | --- | --- | --- | --- | --- | --- | --- | --- | --- | --- | --- | --- | --- | --- | --- | --- | --- | --- | --- | --- | --- | --- | --- | --- | --- | --- | --- | --- | --- | --- | --- | --- | --- | --- | --- | --- | --- | --- | --- | --- | --- | --- | --- | --- | --- | --- | --- | --- | --- | --- | --- | --- | --- | --- | --- | --- | --- | --- | --- | --- | --- | --- | --- | --- | --- | --- | --- | --- | --- | --- | --- | --- | --- | --- | --- | --- | --- | --- | --- | --- | --- | --- | --- | --- | --- | --- | --- | --- | --- | --- | --- | --- | --- | --- | --- | --- | --- | --- | --- | --- | --- | --- |
| **Supplementary Table 6**. Activation peaks for anomalous > pseudoword phrases in the explicit task |
| \| **Region** \| **Cluster size (voxels/mm ³)** \| **x** \| **y** \| **z** \| **T** \| \| --- \| --- \| --- \| --- \| --- \| --- \| \| **L aIFG, DMPFC** \| 3690/57656 \|  \|  \|  \|  \| \| L aIFG (pars orbitalis) \|  \| -42 \| 28 \| -12 \| 8.86 \| \| L posterior-medial frontal gyrus \|  \| -2 \| 18 \| 50 \| 8.71 \| \| L aIFG (pars triangularis) \|  \| -52 \| 36 \| 10 \| 7.91 \| \| L aIFG (pars orbitalis) \|  \| -37 \| 36 \| -15 \| 7.48 \| \| L superior medial gyrus \|  \| -10 \| 38 \| 50 \| 7.28 \| \| **R cerebellum** \| 679/10609 \|  \|  \|  \|  \| \| R cerebellum (crus I) \|  \| 36 \| -72 \| -42 \| 7.21 \| \| R cerebellum (crus II) \|  \| 26 \| -74 \| -48 \| 6.21 \| \| R cerebellum (crus I) \|  \| 18 \| -77 \| -28 \| 6.11 \| \| R cerebellum (crus II) \|  \| 23 \| -82 \| -42 \| 5.56 \| \| R cerebellum (crus I) \|  \| 28 \| -72 \| -28 \| 4.33 \| \| **L/R thalamus, caudate nucleus** \| 422/6593 \|  \|  \|  \|  \| \| R caudate nucleus \|  \| 13 \| 8 \| 12 \| 6.07 \| \| L thalamus \|  \| -4 \| -22 \| 8 \| 5.3 \| \| L thalamus \|  \| -7 \| -12 \| 8 \| 4.9 \| \| L caudate nucleus \|  \| -12 \| 10 \| 10 \| 4.86 \| \| L thalamus \|  \| -12 \| -2 \| 12 \| 4.78 \| \| **L MTG/ITG** \| 365/5703 \|  \|  \|  \|  \| \| L pMTG \|  \| -62 \| -47 \| -2 \| 6.17 \| \| L pMTG \|  \| -50 \| -37 \| -2 \| 5.34 \| \| L pITG \|  \| -52 \| -52 \| -10 \| 4.6 \| \| L pITG \|  \| -50 \| -62 \| -10 \| 4.14 \| \| L pMTG \|  \| -60 \| -62 \| 2 \| 3.18 \| \| **L AG/SMG** \| 225/3515 \|  \|  \|  \|  \| \| L AG \|  \| -40 \| -57 \| 25 \| 5.7 \| \| L AG (PGp) \|  \| -44 \| -70 \| 25 \| 5.3 \| \| **L fusiform gyrus** \| 164/2562 \|  \|  \|  \|  \| \| L fusiform gyrus \|  \| -32 \| -30 \| -25 \| 6.1 \| \| L fusiform gyrus \|  \| -42 \| -17 \| -22 \| 5.39 \| \| L fusiform gyrus \|  \| -40 \| -27 \| -18 \| 4.95 \| \| **L ATL, temporal pole, parahippocampal gyrus** \| 137/2140 \|  \|  \|  \|  \| \| L ATL \|  \| -47 \| -4 \| -32 \| 5.62 \| \| L temporal pole \|  \| -32 \| 6 \| -42 \| 4.64 \| \| L ATL \|  \| -42 \| -4 \| -40 \| 4.06 \| \| L temporal pole \|  \| -47 \| 10 \| -30 \| 3.82 \| \| L temporal pole \|  \| -42 \| 3 \| -32 \| 3.62 \| \| **R aIFG, insula** \| 133/2078 \|  \|  \|  \|  \| \| R insula \|  \| 36 \| 20 \| -8 \| 5.25 \| \| R insula \|  \| 33 \| 26 \| 2 \| 3.77 \| \| R aIFG (pars orbitalis) \|  \| 28 \| 26 \| -12 \| 3.66 \| \| R aIFG (pars orbitalis) \|  \| 43 \| 28 \| -12 \| 3.11 \| \| **R aIFG** \| 51/796 \|  \|  \|  \|  \| \| R aIFG (pars orbitalis) \|  \| 36 \| 38 \| -8 \| 4.84 \| \| R aIFG (pars orbitalis) \|  \| 48 \| 40 \| -12 \| 3.68 \| \| **R amygdala** \| 41/640 \|  \|  \|  \|  \| \| R amygdala \|  \| 28 \| -4 \| -12 \| 5.12 \| \| R pallidum \|  \| 20 \| -2 \| -2 \| 3.42 \| \| **L thalamus** \| 32/500 \|  \|  \|  \|  \| \| L thalamus \|  \| -30 \| -14 \| -8 \| 3.83 \| \| L thalamus \|  \| -24 \| -22 \| -8 \| 3.17 \| \| **brainstem** \| 27/421 \|  \|  \|  \|  \| \| brainstem \|  \| -4 \| -27 \| -25 \| 4.57 \| \| **L ATL** \| 25/390 \|  \|  \|  \|  \| \| L ATL \|  \| -54 \| 3 \| -20 \| 4.3 \| \| **R cerebellum** \| 22/343 \|  \|  \|  \|  \| \| R cerebellum (lobule IX) \|  \| 3 \| -57 \| -50 \| 4.19 \| \| R cerebellum (lobule IX) \|  \| 8 \| -64 \| -42 \| 3.48 \| \| **R cerebellum** \| 22/343 \|  \|  \|  \|  \| \| R cerebellum (crus II) \|  \| -32 \| -77 \| -42 \| 3.59 \| \| **R thalamus** \| 21/328 \|  \|  \|  \|  \| \| R thalamus \|  \| 18 \| -20 \| 0 \| 4.64 \| |

**Supplementary Table 7.** Activation peaks for conjunction of [explicit: meaningful > pseudowords] ∩ [explicit: anomalous > pseudowords]

| **Region** | **Cluster size (voxels/mm³)** | **x** | **y** | **z** | **T** |
| --- | --- | --- | --- | --- | --- |
| **L aIFG, DMPFC** | 2679/41859 |  |  |  |  |
| L aIFG (pars orbitalis) |  | -47 | 40 | -12 | 7.05 |
| L aIFG (pars orbitalis) |  | -34 | 33 | -12 | 6.58 |
| L superior medial gyrus |  | -12 | 38 | 50 | 6.39 |
| L superior medial gyrus |  | -14 | 36 | 55 | 6.28 |
| L aIFG (pars orbitalis) |  | -47 | 46 | -5 | 6.24 |
| **R cerebellum** | 523/8171 |  |  |  |  |
| R cerebellum (Crus II) |  | 38 | -74 | -42 | 6.52 |
| R cerebellum (Crus I) |  | 13 | -80 | -25 | 5.8 |
| R cerebellum (Crus II) |  | 18 | -87 | -38 | 4.74 |
| R cerebellum (Crus II) |  | 20 | -84 | -40 | 4.71 |
| R cerebellum (VIII) |  | 26 | -74 | -48 | 4.28 |
| **L/R thalamus, caudate nucleus** | 357/5578 |  |  |  |  |
| L caudate nucleus |  | -12 | 10 | 10 | 4.86 |
| L caudate nucleus |  | -12 | 0 | 12 | 4.63 |
| L thalamus |  | -2 | -22 | 8 | 4.42 |
| L caudate nucleus |  | -10 | 13 | 2 | 4.31 |
| R caudate nucleus |  | 13 | 10 | 10 | 4.18 |
| **L pMTG/ITG** | 333/5203 |  |  |  |  |
| L pMTG |  | -62 | -47 | -2 | 6.17 |
| L pITG |  | -52 | -52 | -10 | 4.6 |
| L pITG |  | -54 | -57 | -8 | 4.32 |
| L pITG |  | -50 | -62 | -10 | 4.14 |
| L pMTG |  | -60 | -62 | 2 | 3.18 |
| **L AG/SMG** | 224/3500 |  |  |  |  |
| L AG |  | -40 | -57 | 25 | 5.58 |
| L SMG (PFm) |  | -42 | -60 | 22 | 5.56 |
| L AG (PGp) |  | -44 | -70 | 25 | 5.3 |
| **L ATL** | 112/1750 |  |  |  |  |
| L ATL |  | -47 | -4 | -32 | 4.69 |
| L ATL |  | -32 | 3 | -40 | 3.72 |
| L ATL |  | -44 | 8 | -32 | 3.59 |
| L ATL |  | -30 | 0 | -35 | 3.09 |
| L pITG |  | -50 | 10 | -30 | 3.08 |
| **L ITG/fusiform** | 102/1593 |  |  |  |  |
| L fusiform gyrus |  | -30 | -32 | -20 | 5.29 |
| L fusiform gyrus |  | -32 | -30 | -22 | 5.19 |
| L fusiform gyrus |  | -27 | -40 | -18 | 4.04 |
| L fusiform gyrus |  | -42 | -24 | -20 | 3.66 |
| **R insula/IFG** | 83/1296 |  |  |  |  |
| R Insula |  | 36 | 20 | -8 | 5.25 |
| R IFG (pars orbitalis) |  | 36 | 23 | -15 | 3.41 |

**Supplementary Table 8.** Activation peaks for meaningful > pseudoword phrases in the implicit task

| **Region** | **Cluster size (voxel/mm³)** | **x** | **y** | **z** | **T** |
| --- | --- | --- | --- | --- | --- |
| **L AG** | 262/4093 |  |  |  |  |
| L AG (PGp) |  | -50 | -70 | 28 | 6.91 |
| L AG |  | -42 | -57 | 18 | 5.89 |
| L AG (PGp) |  | -52 | -67 | 20 | 5.41 |
| **L DMPFC** | 62/968 |  |  |  |  |
| L middle frontal gyrus |  | -30 | 26 | 42 | 5.05 |
| L superior frontal gyrus |  | -20 | 38 | 42 | 4.65 |
| L superior frontal gyrus |  | -12 | 43 | 45 | 4.54 |
| **L pITG/pMTG** | 34/531 |  |  |  |  |
| L pITG |  | -52 | -30 | -18 | 4.94 |
| L pMTG |  | -62 | -22 | -15 | 4.74 |
| **R ACC** | 23/369 |  |  |  |  |
| R ACC |  | 3 | 30 | -5 | 4.92 |

**Supplementary Table 9**. Activation peaks for conjunction of [explicit: meaningful > pseudowords] & [implicit: meaningful > pseudowords]

| **Region** | **Cluster size (voxel/mm³)** | **x** | **y** | **z** | **T** |
| --- | --- | --- | --- | --- | --- |
| **AG (PGp), pSMG** | 259/4046 |  |  |  |  |
| L AG (PGp) |  | -47 | -70 | 28 | 6.22 |
| L AG (PGp) |  | -40 | -74 | 28 | 5.23 |
| L AG (PGp) |  | -52 | -64 | 20 | 5.01 |
| L AG |  | -42 | -57 | 22 | 4.9 |
| L SMG (PFcm) |  | -50 | -47 | 30 | 4.35 |
| **L pITG** | 21/328 |  |  |  |  |
| L pITG |  | -60 | -37 | -20 | 3.54 |
| L pITG |  | -52 | -32 | -18 | 3.27 |
| **L DMPFC** | 20/312 |  |  |  |  |
| L superior frontal gyrus |  | -17 | 43 | 42 | 4.6 |
| L superior frontal gyrus |  | -12 | 43 | 45 | 4.53 |

**Supplementary Table 10**. Interaction: Explicit > Implicit task for meaningful > pseudoword phrases (inclusively masked with significant voxels from the explicit task: meaningful > pseudoword).

| **Region** | **Cluster size (voxels/mm³)** | **x** | **y** | **z** | **T** |
| --- | --- | --- | --- | --- | --- |
| **L aIFG, insula** | 1132/17688 |  |  |  |  |
| L insula |  | -32 | 20 | 0 | 9.12 |
| L IFG (pars triangularis) |  | -52 | 26 | 25 | 6.69 |
| L insula |  | -27 | 20 | -10 | 6.26 |
| L IFG (pars triangularis) |  | -47 | 33 | 18 | 6.13 |
| L precentral gyrus |  | -40 | 6 | 42 | 6.08 |
| **L/R thalamus, caudate nucleus** | 899/14047 |  |  |  |  |
| R thalamus |  | 13 | -7 | 2 | 5.81 |
| R caudate nucleus |  | 13 | 10 | 8 | 5.75 |
| L caudate nucleus |  | -10 | 13 | 5 | 5.71 |
| L thalamus |  | -10 | -12 | 2 | 5.4 |
| R thalamus |  | 6 | -27 | 5 | 5.27 |
| **L/R ACC, DMPFC** | 697/10891 |  |  |  |  |
| L superior medial frontal gyrus |  | -4 | 28 | 42 | 7.13 |
| R superior medial frontal gyrus |  | 3 | 30 | 38 | 7.02 |
| L superior medial frontal gyrus |  | -2 | 18 | 48 | 6.97 |
| R superior medial frontal gyrus |  | 6 | 23 | 42 | 6.47 |
| L posterior-medial frontal gyrus |  | -2 | 23 | 60 | 5.53 |
| **L IPS/SMG** | 445/6953 |  |  |  |  |
| L SMG (PFt) |  | -44 | -37 | 48 | 6.4 |
| L IPS (hIP3) |  | -40 | -47 | 45 | 6.11 |
| L SMG (PFm) |  | -52 | -60 | 42 | 4.15 |
| L IPS (hIP3) |  | -32 | -60 | 42 | 3.86 |
| L SMG (PFt) |  | -52 | -34 | 45 | 3.85 |
| **L pMTG/ITG** | 227/3547 |  |  |  |  |
| L pITG |  | -47 | -47 | -12 | 4.57 |
| L pITG |  | -57 | -52 | -12 | 4.49 |
| L pITG |  | -57 | -42 | -12 | 4.05 |
| L pMTG |  | -64 | -44 | -5 | 3.45 |
| L pMTG |  | -54 | -40 | -5 | 3.13 |
| **R cerebellum** | 196/3063 |  |  |  |  |
| R cerebellum (crus I) |  | 26 | -67 | -30 | 4.4 |
| R cerebellum (crus II) |  | 26 | -72 | -48 | 4.1 |
| R cerebellum (crus II) |  | 36 | -72 | -48 | 4.07 |
| R cerebellum (crus I) |  | 48 | -62 | -32 | 3.95 |
| R cerebellum (crus I) |  | 43 | -70 | -30 | 3.81 |
| **R aIFG** | 121/1891 |  |  |  |  |
| R IFG (pars orbitalis) |  | 33 | 26 | -5 | 7.85 |
| **R cerebellum** | 78/1219 |  |  |  |  |
| R cerebellum (lobule VI) |  | 10 | -80 | -25 | 4.93 |
| R cerebellum (crus I) |  | 13 | -82 | -32 | 3.82 |
| **R postcentral** | 34/531 |  |  |  |  |
| R S1 |  | 50 | -20 | 58 | 4.24 |
| R S1 |  | 48 | -22 | 48 | 3.38 |
| **L/R ACC** | 30/469 |  |  |  |  |
| L ACC |  | -4 | 6 | 25 | 4.25 |
| R ACC |  | 6 | 8 | 28 | 3.56 |

**Supplementary Table 11.** Interaction: Explicit > implicit task for anomalous > pseudoword phrases (inclusively masked with significant voxels from the explicit task: anomalous > pseudoword).

| **Region** | **Cluster size (voxels/mm³)** | **x** | **y** | **z** | **T** |
| --- | --- | --- | --- | --- | --- |
| **L aIFG, insula** | 1186/18531 |  |  |  |  |
| L IFG (pars triangularis) |  | -52 | 33 | 10 | 6.51 |
| L insula |  | -27 | 26 | 2 | 6.46 |
| L IFG (pars triangularis) |  | -54 | 20 | 25 | 6.41 |
| L insula |  | -37 | 20 | -2 | 6.2 |
| L IFG (pars triangularis) |  | -47 | 23 | 0 | 5.92 |
| **L DMPFC** | 639/9984 |  |  |  |  |
| L posterior-medial frontal gyrus |  | -2 | 18 | 50 | 7.26 |
| L superior medial frontal gyrus |  | -2 | 28 | 42 | 5.84 |
| L posterior-medial frontal gyrus |  | 0 | 18 | 60 | 5.76 |
| L superior medial frontal gyrus |  | 0 | 36 | 50 | 4.81 |
| L superior medial frontal gyrus |  | -7 | 36 | 40 | 4.35 |
| **R cerebellum** | 141/2203 |  |  |  |  |
| R cerebellum (lobule VI) |  | 28 | -60 | -32 | 4.63 |
| R cerebellum (crus I) |  | 46 | -60 | -32 | 4.52 |
| R cerebellum (lobule VIIb) |  | 23 | -72 | -48 | 4.48 |
| R cerebellum (crus I) |  | 36 | -70 | -40 | 4.42 |
| R cerebellum (lobule VI) |  | 28 | -60 | -32 | 4.63 |
| **R thalamus/caudate nucleus** | 112/1750 |  |  |  |  |
| R thalamus |  | 13 | -17 | 12 | 5.07 |
| R caudate nucleus |  | 16 | 6 | 12 | 5.03 |
| R thalamus |  | 8 | -4 | 5 | 4.57 |
| **R aIFG** | 102/1593 |  |  |  |  |
| R IFG (pars orbitalis) |  | 33 | 26 | -5 | 7.12 |
| **R cerebellum** | 81/1265 |  |  |  |  |
| R cerebellim (lobule VI) |  | 10 | -77 | -25 | 5.41 |
| **L thalamus** | 67/1046 |  |  |  |  |
| L thalamus |  | -14 | -20 | 12 | 4.66 |
| L thalamus |  | -4 | -22 | 8 | 4.21 |
| **L caudate nucleus** | 35/546 |  |  |  |  |
| L caudate nucleus |  | -14 | 3 | 15 | 4.17 |
| L caudate nucleus |  | -12 | 8 | 8 | 4.02 |

**Supplementary Table 12.** Activation peaks for the psychophysiological interaction during the explicit task for meaningful > pseudowords seeded from the left PGp

| **Region** | **Cluster size (voxel/mm³)** | **x** | **y** | **z** | **T** |
| --- | --- | --- | --- | --- | --- |
| **Pre-SMA** | 106/1656 |  |  |  |  |
| L pre-SMA |  | -4 | 6 | 6 | 5.2 |
| R SMA |  | 8 | 0 | 60 | 4.16 |
| **L aIFG** | 73/1140 |  |  |  |  |
| L aIFG (pars triangularis) |  | -54 | 20 | 8 | 4.93 |
| L aIFG (pars triangularis) |  | -40 | 20 | 5 | 4.18 |
| L aIFG (pars orbitalis) |  | -44 | 23 | -5 | 4.03 |
